# Changes in Hippocampal Volume are Correlated with Cell Loss but Not with Seizure Frequency in Two Chronic Models of Temporal Lobe Epilepsy

**DOI:** 10.3389/fneur.2014.00111

**Published:** 2014-07-01

**Authors:** Roberson S. Polli, Jackeline M. Malheiros, Renan dos Santos, Clement Hamani, Beatriz M. Longo, Alberto Tannús, Luiz E. Mello, Luciene Covolan

**Affiliations:** ^1^Departamento de Fisiologia, Universidade Federal de São Paulo – UNIFESP, São Paulo, Brazil; ^2^Centro de Imagens e Espectroscopia in vivo por Ressonância Magnética (CIERMag), Instituto de Física de São Carlos, Universidade de São Paulo (IFSC-USP), São Carlos, Brazil; ^3^Departamento de Farmacologia, Universidade Federal de São Paulo – UNIFESP, São Paulo, Brazil; ^4^Division of Neurosurgery, Toronto Western Hospital, University of Toronto, Toronto, ON, Canada

**Keywords:** kainic acid, pilocarpine, magnetic resonance imaging, epilepsy

## Abstract

Kainic acid (KA) or pilocarpine (PILO) have been used in rats to model human temporal lobe epilepsy (TLE) but the distribution and severity of structural lesions between these two models may differ. Magnetic resonance imaging (MRI) studies have used quantitative measurements of hippocampal T_2_ (T_2_HP) relaxation time and volume, but simultaneous comparative results have not been reported yet. The aim of this study was to compare the MRI T_2_HP and volume with histological data and frequency of seizures in both models. KA- and PILO-treated rats were imaged with a 2 T MRI scanner. T_2_HP and volume values were correlated with the number of cells, mossy fiber sprouting, and spontaneous recurrent seizures (SRS) frequency over the 9 months following status epilepticus (SE). Compared to controls, KA-treated rats had unaltered T_2_HP, pronounced reduction in hippocampal volume and concomitant cell reduction in granule cell layer, CA1 and CA3 at 3 months post SE. In contrast, hippocampal volume was unchanged in PILO-treated animals despite detectable increased T_2_HP and cell loss in granule cell layer, CA1 and CA3. In the following 6 months, MRI hippocampal volume remained stable with increase of T_2_HP signal in the KA-treated group. The number of CA1 and CA3 cells was smaller than age-matched CTL group. In contrast, PILO group had MRI volumetric reduction accompanied by reduction in the number of CA1 and CA3 cells. In this group, T_2_HP signal was unaltered at 6 or 9 months after status. Reductions in the number of cells were not progressive in both models. Notably, the SRS frequency was higher in PILO than in the KA model. The volumetry data correlated well with tissue damage in the epileptic brain, suggesting that MRI may be useful for tracking longitudinal hippocampal changes, allowing the assessment of individual variability and disease progression. Our results indicate that the temporal changes in hippocampal morphology are distinct for both models of TLE and that these are not significantly correlated to the frequency of SRS.

## Introduction

Temporal lobe epilepsy (TLE) is frequently associated with hippocampal neuronal loss, synaptic reorganization, and mesial temporal sclerosis (1–3). Several animal models, including those following systemic injections of kainic acid (KA) or pilocarpine (PILO), have been developed to study the underlying mechanisms of TLE in rodents. In both models, after the status epilepticus (SE), animals develop spontaneous recurrent seizures (SRS), and neuropathological alterations that resemble human TLE (4–9). The comparative histopathology after SE indicates that the KA group is more likely to develop and to survive SE, while the PILO group shows a greater number of brain areas affected at a high intensity within a shorter time interval (6). In preclinical models of epilepsy, volumetric magnetic resonance imaging (MRI) studies have shown a significant atrophy of limbic structures as well as T_2_ changes that correlate with pathological states (10–16). However, there are no reports comparing the long-term temporal changes that occur in the hippocampus after injections of KA with those after injections of PILO.

Therefore, we conducted a longitudinal study in chronic epileptic rats to investigate hippocampal volumetric and T_2_ changes 3, 6 and 9 months following KA- or PILO-induced SE. The recorded MRI values were correlated with the degree of cell loss in various hippocampal regions, mossy fiber sprouting, and the frequency of SRS.

## Experimental Procedures

### Animal models

All protocols were approved by the Animal Care Committee of the Universidade Federal de São Paulo. Adult male Wistar rats (250–300 g) were systemically injected with either kainic acid (10 mg/kg i.p., Sigma, Saint Louis, MI, USA) or pilocarpine hydrochloride (320 mg/kg, i.p. Vegeflora, Parnaiba, Brazil). Thirty minutes prior to PILO administration, animals were given scopolamine methyl bromide (1 mg/kg, i.p., Sigma, Saint Louis, MI, USA) to reduce systemic cholinergic side effects. Animals in the KA and PILO groups developed SE on average 90 and 30 min after the injections, respectively. SE was defined as a state of continuous seizures lasting longer than 5 min (17, 18). To reduce the associated mortality rate and to balance the length of SE, a single dose of thionembutal (25 mg/kg, i.p., Cristalia, Sao Paulo, Brazil) was given to all animals 90 min following SE onset. All rats were closely monitored during and in the hours after SE. Animals that did not exhibit SE were considered resistant, and they were excluded from the study. Saline-treated controls (CTL, *n* = 18) were injected with thionembutal but not with KA (*n* = 38) or PILO (*n* = 45). The MRI data were obtained from 59 animals: KA (*n* = 22), PILO (*n* = 22), and CTL (*n* = 15). The SE-related mortality was 28% for the PILO group and 5% for the KA group. During the 1-year study, 31% of the animals in the PILO group, 39% in the KA group, and 18% in the CTL group died, and they were excluded from the analysis.

### Frequency of seizures

Seizures were monitored by video recording beginning 45 days after SE and continuing until sacrifice (12 h/day, 5 days/week). The 12-h schedule was randomized to record animals during both light and dark phases. To record seizures in the dark, infrared light was used to illuminate the cages. Animals were housed in acrylic cages (one animal per cage) to allow optimal video observation. The severity of the SRS was graded in stages, as previously described (19). Only the following stages were considered SRS: III (bilateral forelimb clonus), IV (rearing), or V (loss of postural tone, i.e., rearing and falling). The duration of each SRS was obtained from video recordings because this method was previously shown to be as accurate as EEG recordings for the analysis of generalized seizures (≥stage III) (20), when large number of animals are simultaneously recorded.

### Magnetic resonance imaging

Animals were scanned 3 (KA: *n* = 22; PILO: *n* = 22; CTL: *n* = 15), 6 (KA: *n* = 16; PILO: *n* = 16; CTL: *n* = 10) and 9 months after SE (KA: *n* = 9; PILO: *n* = 9; CTL: *n* = 5). At each time point, five animals from each group were randomly sacrificed so that we could correlate the MRI results with the histological findings. Images were obtained in a 2 T/30 cm superconducting magnet 85310HR (Oxford Instruments, Abingdon, UK) interfaced with a Bruker Avance AVIII console (Bruker-Biospin, Inc., Billerica, MA, USA) using Paravision 5.0 software (Bruker-Biospin, Inc., Billerica, MA, USA). A crossed saddle radiofrequency coil (Papoti, 2006) was used as a head probe in animals anesthetized with ketamine/xylazine (95/12 mg/kg, i.p.). For volumetry, a T_2_-weighted Rapid Acquisition with Refocused Echoes (RARE) sequence was used (TR = 4500 ms, TE = 67.1 ms, RARE factor = 6, 18 averages, 45 min/animal). A volume of 40 mm × 40 mm × 11.2 mm was covered with a 192 × 192 matrix and 700-μm-thick slices without gaps (16 slices), generating a spatial resolution of 208 μm × 208 μm. Thereafter, a T_2_ Multi-Slice Multi-Echo (MSME) sequence was acquired with similar spatial resolution to determine the hippocampal T_2_ relaxation time (average of four runs, TR = 2000 ms, 15 equally spaced echoes, TE = 15–225 ms, 19 min/animal). The parameters for the T_2_ MSME sequence were chosen to reduce the anesthesia time because longer times could increase the mortality rate in chronic epileptic animals.

The MRI data were analyzed using Paravision 5.0 software. One author (RSP), blinded to the identity of the groups, manually outlined the hippocampus, and intracranial vault. For volumetric analyses, the regions of interest (ROI) representing the hippocampus were assessed in six regularly separated T_2_-weighted coronal sections, from 2.1 to 6.1 mm caudal to bregma as previously described (15). Measurements from both hemispheres were matched. The three most rostral coronal levels (2.1, 3.0, and 3.9 mm caudal to bregma) were considered the rostral hippocampus (RH), and the three most caudal levels (4.8, 5.2, and 6.1 mm caudal to bregma) were considered the caudal hippocampus (CH) (15). Hippocampal atrophy was defined as a value lower than the mean value minus the standard deviation measured in the control group.

The sequence for relaxometry (MSME) was acquired according to the protocol described above. The ROI was drawn on T_2_-weighted images (approximately 3.0 mm caudal to bregma) and incorporated into relaxometry images by using Paravision software. The ISA (Image Sequence Analysis) software was used, and the T_2_ relaxation time calculated from a monoexponential curve.

### Histological and stereological analysis

One week after each MRI scan (3, 6 or 9 months after SE), five animals from each group were anesthetized with chloral hydrate (1 mg/kg, i.p.) and transcardiacally perfused for histological analysis of cell loss and of mossy fiber sprouting in the hippocampal formation. These animals were randomly chosen by an author (JMM) who was unaware of the frequency of spontaneous seizures in the animals. For perfusion, the following sequence of solutions was used: (1) 100 ml of phosphate buffer (2), 250 ml of fixative sulfide 0.1% sodium Millonig’s buffer (3), and 500 ml of paraformaldehyde. Thereafter, the brains were removed from the skulls, immersed in 30% sucrose overnight and cut on a cryostat (40 μm-thick sections). A one-in-three series of consecutive sections was stained by the neo-Timm histological method (21) to detect mossy fiber sprouting (22). The staining was performed with the following solutions: 240 ml of 50% gum Arabic with 10.25 g of citric acid, 9.45 g sodium citrate in 30 ml of ddH_2_O, 3.73 g hydroquinone in 60 ml of ddH_2_O, and 2 ml of 0.51 g silver nitrate in 3 ml ddH_2_O. Sections were developed for 45 min, washed twice in distilled water for 5 min, dehydrated through a series of alcohols followed by xylene, and protected with coverslips. A series of adjacent sections was processed for Nissl staining for later use in cell counting.

Images from histological sections (10× objective lens; Nikon microscope Eclipse E600FN) were captured with a high-resolution digital camera (Nikon DXM1200) and converted into digital signals that were transmitted to a computer. The intensity of sprouting in the dentate supragranular layer was evaluated by using quantitative measures obtained from gray scale values with the NIH Image J software (http://rsbweb.nih.gov/ij/index.html). Gray scale values for the supragranular layer were compared to those obtained for the corpus callosum (baseline).

One hemisphere was selected at random for estimating the number of cells. The stereological analysis of Nissl-stained cells was performed using the Stereo Investigator software (version 9.13, MicroBrightField, USA). The optical fractionator method (23) was used to estimate the total number of cells in each subregion, which were analyzed at a high magnification with a 100× oil objective. This method is independent of volume measurements and thus unaffected by tissue shrinkage. The sampling of tissue sections followed a systematic, uniform and random scheme to ensure an equal probability of sampling from all parts of the structure. Thus, the analysis of the sections started from a random position at the origin of the brain structures. Every sixth section was included. A total of 11–13 sections containing the hippocampal subregions (CA1, CA3, dentate granule cell layer and hilus) were used for stereological examination. The contour delineations of the subregions were defined according to an atlas (24) and were drawn using a 4× objective.

An investigator blinded (RS) to treatment group has counted Nissl-stained neurons in the granule cell layer, hilus, and pyramidal cell layer of CA3 and CA1. The hilus region was defined by its border with the granule cell layer and straight lines drawn from the tips of the granule cell layer to the proximal end of the CA3 pyramidal cell layer. All cells enclosed within this area were counted. The border between the CA3 and CA2 pyramidal cell layer was determined by the distal end of mossy fibers, labeled in adjacent Timm-stained sections. CA1 was identified by a single continuous layer of pyramidal cells. Subicular and CA2 neurons were not counted. Glial cells, characterized by smaller size (<3 μm) as well as peculiar cytological features (dense bodies and large nuclei surrounded by a sparse cytoplasm) were not included in the estimates.

After a pilot test evaluating the stereological counting method, each counting frame (30 μm × 30 μm for the CA1, CA3 and dentate gyrus, and 40 μm × 40 μm for the hilus) was placed at an intersection of the lines forming a virtual grid (250 μm × 150 μm) that was randomly placed by the software within each subregion of interest. The optical dissector height (thickness) was 12 μm with a 1-μm top and bottom guard zone. The mean section thickness measured at every other counting frame site was used for the final calculation of cell number for each subregion analyzed. The estimation of cell number was calculated using the total number of cells counted at the dissectors (*Q^−^*), the section sampling fraction (*ssf*, number of sections sampled per total sections), the area sampling fraction (*asf*, area of section sampled per total area), and the height sampling fraction (*hsf*, section thickness per dissector height). Thus, the total cell population can be estimated from: $N=\frac{1}{ssf}\cdot \frac{1}{asf}\cdot \frac{1}{hsf}\cdot \Sigma Q-$, where *N* is the estimated total number of cells in the structure analyzed, and ΣQ− is the total number of nuclei counted. As the estimation of cell number is variable according to species, sex, age [for review see Ref. (25)], the precision of the individual estimations, which is expressed by a coefficient of error (CE), which was set at ≤0.03.

The Cavalieri estimator probe method (26, 27) was used to obtain the histological cell volume and this was compared to the volumetric data obtained using MRI and the cell number obtained using stereological cell counting methods. In brief, an Olympus microscope coupled with a camera was connected to a personal computer running the Stereo Investigator software. Stereological analysis was conducted throughout the rostral–caudal extent of the hippocampus. One out of every 12 sections – corresponding to the number of slices measured for MRI – was randomly chosen (six slices per animal). The contour of the structures was drawn around the region corresponding to the hippocampus in each section. A grid (150 μm × 150 μm) was superimposed over the sections, and all points lying within the counter were automatically recorded. Using the Cavalieri method (26), the volume (V) of each region was estimated as V = TaΣP_i_, where *T* is the mean slice thickness, *a* is the area per point and ΣP_i_ is the sum of points hitting the marked region. Coefficients of error were calculated, and values <0.10 were accepted. The number of cells is expressed as number of cells × 1000.

### Statistical analysis

Results are expressed as the mean ± SEM. The comparisons across the three groups were conducted using a one-way ANOVA (Bonferroni *post hoc*). Pearson’s correlation coefficient (*r*) was derived to investigate the potential relationships among the data obtained from MRI volumetry/relaxometry, neo-Timm densitometry, stereological cell counting, and seizure rates. The results were considered statistically significant when *P* < 0.05.

## Results

### Magnetic resonance imaging

**Figure 1:**
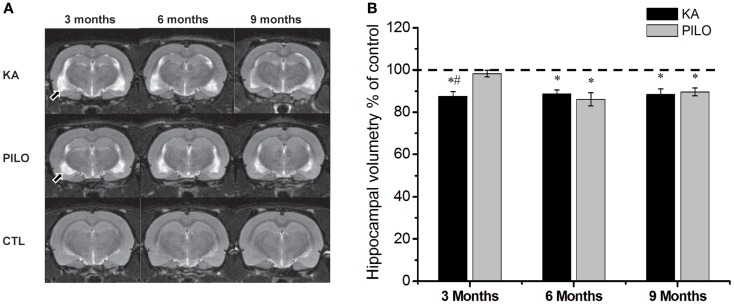
**Hippocampal MRI volumetry with quantitative measurements from rats treated with kainic acid (KA) or pilocarpine (PILO) in animal models of chronic epilepsy**. In **(A)**, representative MRI scans of animals from KA, PILO, and control (CTL) groups at 3, 6, or 9 months post status epilepticus (SE). In **(B)**, the temporal evolution of hippocampal volumetric changes in both experimental models. At 3 months post SE, the KA group shows a significant reduction in hippocampal volumetric as compared to the PILO (^#^*P* < 0.01) and control groups (*P < 0.05). Both models, however, had a similar profile at 6 and 9 months post SE. Data is expressed as a percentage of control. Arrows in **(A)** indicate ventricular changes in the KA and PILO groups compared to controls.

Our hippocampal MRI volumetric findings are summarized in Figure 1. Three months after SE, KA-treated animals had a significant decrease in hippocampal volume (60.9 ± 1.5 mm^3^) compared with PILO-treated rats (68.3 ± 1.1 mm^3^; *P* = 0.0010) or with saline-treated controls (69.6 ± 0.6 mm^3^; *P* = 0.00005). Hippocampal atrophy was present in 90% of the animals in the KA group and in 70% of the animals in the PILO group, 3 months post SE. Differences across groups were recorded equally in the RH and in the CH (Table 1). Six months after SE, hippocampal volumes in both the KA- (66.2 ± 1.3 mm^3^) and PILO-treated rats (64.2 ± 2.3 mm^3^) were smaller than those in the controls (74.7 ± 0.9 mm^3^, *P* = 0.01064 and *P* = 0.00133, respectively). At this time point post SE, the hippocampal atrophy remained evident in 90% of KA-treated animals, but it increased to 82% rats in the PILO-treated group. Even when the RH and CH were considered separately, the volume differences between the KA- and PILO-treated rats were still detected at this time point (Table 1; Figure 1). Nine months after SE, hippocampal volumes in the KA (66.9 ± 1.9 mm^3^) or PILO (67.8 ± 1.4 mm^3^) groups were similar and smaller than those in the control group (75.7 ± 1.4 mm^3^; *P* = 0.00795 and *P* = 0.01287, respectively) (Figure 1). From the acquisition of the first image (3 months after SE) to the last image (9 months after SE), the hippocampal volume in the KA group remained stable, while at the last image acquisition, 88% of the rats in the PILO group had hippocampal atrophy (~20% increase). In summary, rats in the KA group had pronounced hippocampal atrophy in the first 3 months post SE and remained relatively stable during the following 6 months. In contrast, hippocampal atrophy was detected later in the PILO group and was more pronounced in the acquisition made at 6 months post SE than at 3 months post SE. Thereafter, it remained relatively stable.

**Table 1:** **Hippocampal MRI volumetry**.

	KA		PILO		CTL	
	RH	CH	RH	CH	RH	CH
3 months	13.4 ± 0.5*^#^	47.5 ± 1.3*^#^	16.6 ± 0.4^#^	51.7 ± 0.9^#^	17.5 ± 0.5	52.1 ± 0.4
6 months	14.9 ± 0.5*	51.3 ± 1.2	14.5 ± 0.5*	49.6 ± 1.8*	18.7 ± 0.5	55.9 ± 0.9
9 months	13.7 ± 0.7*	53.2 ± 1.5*	15.5 ± 0.5	52.4 ± 1.1*	17.5 ± 0.8	58.2 ± 0.8

*P < 0.05 between the experimental group and control.

*^#^*P* < 0.05 between experimental groups*.

**Figure 2:**
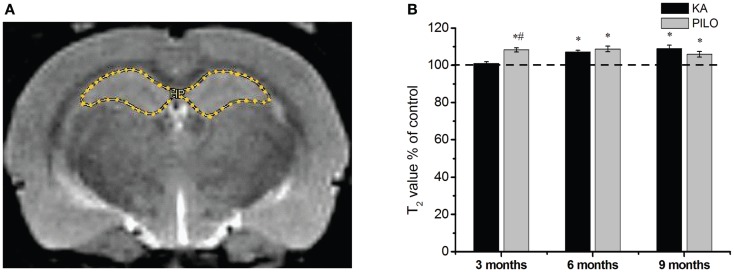
**Hippocampal MRI relaxometry and quantitative measurements from chronic epileptic rats treated with kainic acid (KA) or pilocarpine (PILO)**. In **(A)**, a representative control image highlights the hippocampal region of interest (ROI) for relaxometry analysis. In **(B)**, 3 months after status epilepticus (SE), PILO-treated animals show a T_2_ increase compared with KA-treated (^#^*P* < 0.01) and control groups (*P < 0.05). This increase remained stable throughout the 9 months period of observation. The KA group shows T_2_HP increases 6 months after SE (*P < 0.05 compared with control), which remained stable thereafter. Data is expressed as a percentage of controls.

Our hippocampal MRI relaxometry findings are summarized in Figure 2. All KA- or PILO-treated animals showed T_2_ alterations. Three months after SE, T_2_HP values were higher for the animals in the PILO group (65.4 ± 0.7 ms) when compared with those values for animals in the KA (61.3 ± 0.5 ms, *P* = 0.00021) or control groups (60.7 ± 0.8 ms, *P* = 0.00006). Six months after SE, T_2_HP values recorded in both KA- (62.4 ± 0.6 ms) and PILO-treated rats (63.4 ± 0.9 ms) were higher than those in controls (58.3 ± 1.2 ms; *P* = 0.00213 and *P* = 0.01724, respectively). Nine months after SE, T_2_HP values for KA- (64.5 ± 1.1 ms) and PILO-treated rats (62.7 ± 0.9 ms) were higher than for controls (59.2 ± 0.9 ms; *P* = 0.01407 and *P* = 0.01527, respectively).

### Histology

**Figure 3:**
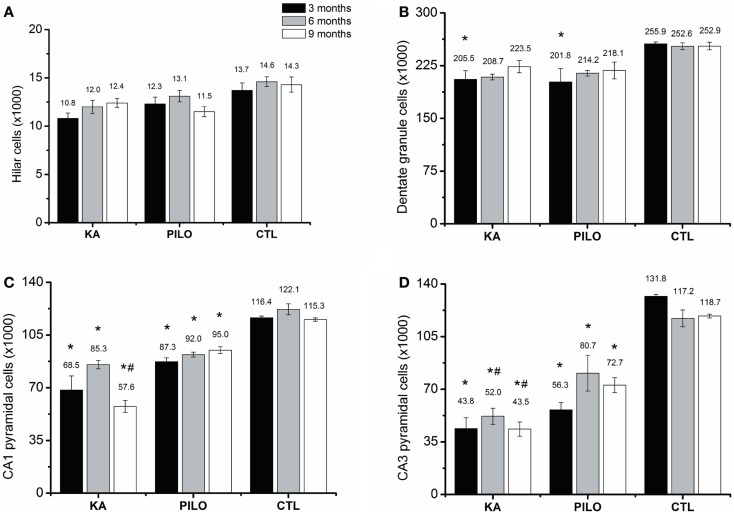
**Hippocampal histological analysis of Nissl-stained sections (number of cells ×1000)**. The number of hilar **(A)** is stable over the time for all groups. Differences in the number of cells are reported by comparing both experimental groups with their age-matched controls (*) or between both experimental groups (^#^). The number of granule cells **(B)** is reduced in both models (*P < 0.05). Three months after status the number of pyramidal CA1 **(C)** and CA3 cells **(D)** was similar between KA and PILO groups, both being reduced when compared with CTL (*P < 0.01). Six months after status, the number of pyramidal CA1 **(C)** is reduced in the KA and PILO groups (*P < 0.01). The same was observed for pyramidal CA3 cells **(D)** (*P < 0.01). At this time point, the number of pyramidal CA3 cells was lower in the KA than PILO group (^#^*P* < 0.05). Nine months after status, KA and PILO groups had reduction in the number of pyramidal CA1 (*P < 0.01 and *P < 0.05, respectively) and CA3 cells (*P < 0.01 for both groups). At this time point, KA had lower number of CA1 (^#^*P* < 0.01) and CA3 cells (^#^*P* < 0.05) than PILO group.

The number of cells as revealed by stereological counting methods using Nissl-stained tissue was recorded in the CA1, CA3, dentate hilus, and granule cell layer of the hippocampus in control, KA- and PILO-treated rats. Detailed examination of each time point reveals that the number of cells was stable in control animals for all hippocampal areas. Similarly, the number of hilar cells was similar over time in both experimental groups (Figure 3A). Differences in the number of cells were revealed by comparing both experimental groups with their age-matched controls. Three months after status, the number of granule (Figure 3B), pyramidal CA1 (Figure 3C), and CA3 cells (Figure 3D) was similar between KA and PILO groups, both reduced when compared to CTL CA1 (*P* = 0.00014 and *P* = 0.00040, respectively) and CA3 cells (*P* = 0.00014). Six months after status, the number of pyramidal CA1 and CA3 cells was reduced in the PILO (*P* = 0.00029 and *P* = 0.00373, respectively) and KA groups (*P* = 0.00014; Figure 3C). At this time point, KA group had even lower number of pyramidal CA3 cells than PILO group (*P* = 0.04197, Figure 3D). Nine months after status, The KA group had a reduction in the number of pyramidal CA1 (*P* = 0.00014, Figure 3C) and CA3 cells (*P* = 0.00014, Figure 3D) as compared to CTL. Similarly, PILO group cell reduction was detected in CA1 (*P* = 0.02376, Figure 3C) and CA3 (*P* = 0.00025, Figure 3D). At this time point, KA group higher cell loss than PILO group in CA1 (*P* = 0.00014, Figure 3C) and in CA3 (*P* = 0.03675, Figure 3D).

**Figure 4:**
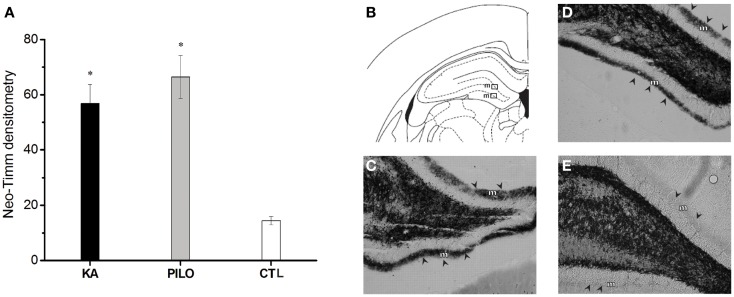
**Graphic representation of neo-Timm densitometry (A)**. The MFS is similar in both experimental groups but different from controls (*P < 0.01). In **(B–E)**: atlas level **(B)** used to represent examples of MFS in the molecular layer (m) of KA **(C)**, PILO **(D)**, and CTL **(E)**. The molecular layer is indicated by arrowheads in **(C–E)**.

The results of the densitometry following staining with the neo-Timm method revealed that mossy fiber sprouting was clearly observed in both epileptic groups (KA: 56.8 ± 6.9; PILO: 66.4 ± 7.7; Figure 4) different from control (14.5 ± 1.5, *P* = 0.00082 and *P* = 0.00001) group.

### Histology correlations with MRI

**Figure 5:**
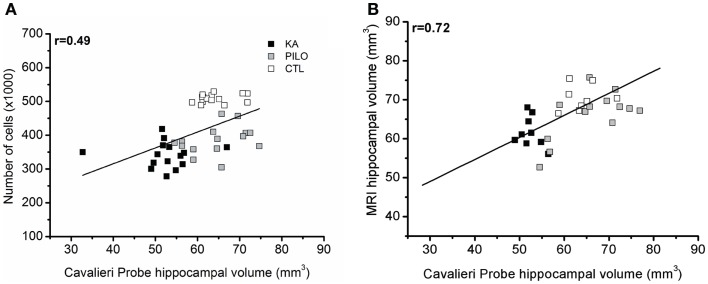
**Cavalieri probe hippocampal volume correlations. (A)** Cavalieri probe hippocampal volume (*x* axis) and number of cells (*y* axis, ×1000) correlation for pooled time groups [*r* = 0.49; *P* = 0.00069]. In **(B)**, the correlation between MRI volumetric values (*y* axis) and Cavalieri probe analysis is shown (*x* axis) (*r* = 0.72; *P* = 0.00001).

Cavalieri probe for hippocampal volume showed positive correlation with stereological counting of number of hippocampal cells (×1000) (*r* = 0.49, *P* = 0.00069; Figure 5A) when the three groups were considered, and *r* = 0.45 (*P* = 0.01225, not shown) when the control group is excluded from analysis. With strong positive correlation, the Cavalieri probe also validated the MRI measurements of hippocampal volume (*r* = 0.72, *P* = 0.00001; Figure 5B).

**Figure 6:**
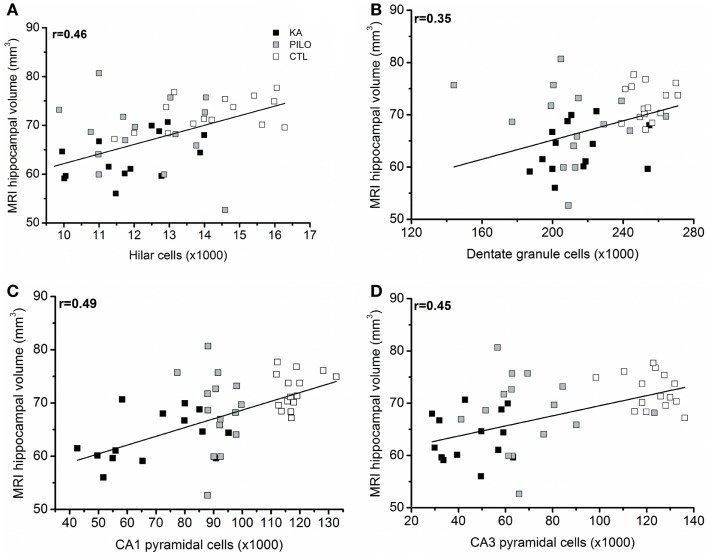
**(A)** correlation between the number of dentate hippocampal cells and the MRI hippocampal volume was positive for the hilus (*r* = 0.46, *P* = 0.00143), granule cell layer [**(B)**, *r* = 0.35, *P* = 0.02007]. Correlation was also positive for pyramidal CA1 **(C)** (*r* = 0.49, *P* = 0.00071) and CA3 cells **(D)** (*r* = 0.45, *P* = 0.00493).

When considering the three groups, the analysis of correlation between the number of dentate hippocampal cells and the MRI hippocampal volume was positive for the hilus (*r* = 0.46, *P* = 0.00143; Figure 6A), granule cell layer (*r* = 0.35, *P* = 0.02007; Figure 6B), pyramidal CA1 (*r* = 0.49, *P* = 0.00071; Figure 6C), and CA3 cells (*r* = 0.45, *P* = 0.00493; Figure 6D). Even when the control group is excluded from analysis, positive correlation was obtained between the number of cells and MRI hippocampal volume for all hippocampal areas, where in the hilus: *r* = 0.43 (*P* = 0.00824); granule cell layer: *r* = 0.37 (*P* = 0.01861); pyramidal CA1 *r* = 0.45 (*P* = 0.00206), and CA3 cells *r* = 0.39 (*P* = 0.00351).

Although no differences were found in animals sacrificed 3, 6, or 9 months after SE, mossy fiber sprouting was slightly greater in those animals with higher cell losses in the CA1 (*r* = 0.49; *P* = 0.00072) and CA3 fields (*r* = 0.52; *P* = 0.00024). No correlation was observed between mossy fiber sprouting and number of cells in the hilus or granule cell layer (data not shown). It is important to recognize that no attempt was made to distinguish different cell populations (as mossy cells, interneurons, or ectopic granule cells) in the hilus that could be differentially related to mossy fiber aberrant reorganization.

### Spontaneous seizure frequency, duration, and correlations

**Figure 7:**
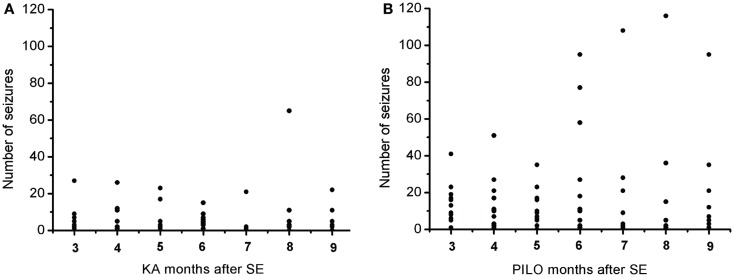
**Graphic representation of the monthly distribution of spontaneous recurrent seizures in rats treated with (A) kainic acid (KA) or (B) pilocarpine (PILO) over the 9 months following status epilepticus (SE)**.

The frequency of the SRS observed for animals in the PILO group was significantly higher than that for rats in the KA group (13.9 ± 2.6 vs. 6.2 ± 1.8 per animal during the total time of observation; *P* = 0.02539; Figure 7 and Table 2). Interestingly, 91% of all PILO-treated rats had SRS in the third month post SE (9.9 ± 2.1 seizures/month), but only 70% of the animals demonstrated hippocampal atrophy. In contrast, at this same time point, 90% of all KA-treated rats demonstrated hippocampal atrophy, but only 72% had SRS (3.7 ± 1.4 seizures/month; *P* = 0.04568 when compared to the PILO group). The seizure rate in epileptic animals was highly variable in both models, especially pilocarpine. Most animals in the KA model have less than 10 seizures a month, even though these appear superposed or not in the Figure 7A. On the other hand, PILO-treated animals tend to have a more widely distributed profile of seizures (Figure 7B). The seizure frequency does not correlate with hippocampal volume (MRI and histological data), T_2_ relaxation time or mossy fiber sprouting. The duration of each spontaneous seizure was comparable in both models (KA = 20.3 ± 3.3 s; PILO = 24.8 ± 2.8 s).

**Table 2:** **Absolute number of spontaneous seizures^a^ after KA- or PILO-induced SE**.

	KA								PILO						
	3 months	4 months	5 months	6 months	7 months	8 months	9 months		3 months	4 months	5 months	6 months	7 months	8 months	9 months
KA 1	2	26	5	3	1	65	22	PILO 1	41	21	23	10	0	5	12
KA 2	1	5	0	4	21	11	5	PILO 2	16	51	35	5	3	2	9
KA 3	1	5	0	7	0	3	2	PILO 3	1	11	2	58	2	15	21
KA 4	2	0	1	6	0	2	0	PILO 4	5	3	17	11	21	36	7
KA 5	0	11	17	9	2	0	3	PILO 5	5	0	7	18	2	1	95
KA 6	2	0	3	1	0	5	11	PILO 6	1	7	5	1	0	0	1
KA 7	0	0	1	1	0	0	3	PILO 7	5	27	16	77	108	116	5
KA 8	1	0	0	0	2	0	3	PILO 8	0	1	0	2	0	0	1
KA 9	5	12	23	15	21	11	6	PILO 9	0	1	9	27	28	5	0
KA 10	0	1	0	3				PILO 10	16	2	0	1			
KA 11	0	0	2	0				PILO 11	19	10	6	0			
KA 12	3	0	2	1				PILO 12	16	21	7	1			
KA 13	7	2	0	5				PILO 13	8	1	10	95			
KA 14	9	25	26	12				PILO 14	5	17	7	11			
KA 15	5	0	23	15				PILO 15	5	1	0	1			
KA 16	27	5	7	10				PILO 16	1	0	0	1			
KA 17	3							PILO 17	9						
KA 18	0							PILO 18	13						
KA 19	11							PILO 19	6						
KA 20	0							PILO 20	5						
KA 21	3							PILO 21	23						
KA 22	27							PILO 22	17						

*^a^Number of video-recorded spontaneous seizures ≥stage III (19) per month, 12 h/day, 5 days/week from 3 to 9 months after status*.

## Discussion

We evaluated MRI volume and signal changes in the hippocampus of KA- or PILO-treated chronic epileptic rats from the third to the ninth month after SE. The major original findings of this study are that post SE, both groups had a time-specific reduction in MRI hippocampal volume and elevation in T_2_ values compared to controls. The volume reductions as assessed by MRI occurred earlier in the KA-treated rats than in the PILO-treated animals, although ultimately both models produced comparable volume reductions. In contrast, MRI signal alterations occurred first in the PILO model of TLE, although the signal changes again were ultimately comparable for the two experimental groups. The number of hippocampal cells in neither epileptic group was correlated with the frequency of SRS. There was also no correlation of seizure frequency with MRI hippocampal volume reduction or with increased T_2_ relaxation times. Taken together, these data support the view that hippocampal alterations are not necessarily related to the frequency of seizures.

One advantage of this comparative study is that subtle temporal differences between models could be detected in the first 3 months post SE. In the KA model of TLE, the change in hippocampal volume as revealed by MRI occurred as early as 3 months following SE, while more time was necessary to detect this change in the PILO model. This indicates that hippocampal volumetric loss in the PILO model takes longer than in the KA model but reaches levels similarly stable over time. Indeed, it is important to emphasize that hippocampal cell loss is not progressive in either model. An alternative explanation for the initial differences (3 months after SE) between the two models may be that the PILO treatment may lead to higher levels of water release (edema) than the KA treatment, resulting in an increased MRI volume and hyperintense T_2_ signal. The atrophy detected by MRI as early as 3 months after SE in KA-treated animals is consistent with the stereological data as well as with the data obtained from Cavalieri’s method; however, the possible differences in edema between the PILO and KA groups will require further investigated. In both models, the hippocampal atrophy did not correlate with the frequency of seizures. The higher cell loss observed in the pyramidal cell fields of the KA group contrasts with the higher frequency of spontaneous seizures observed in the PILO group. Hippocampal volumetric loss was also uncorrelated with supragranular mossy fiber synaptic sprouting, which remained unaltered following the epileptic seizures produced in both models. The key differences described in this report for two models of TLE may be useful when designing specific objectives for future studies.

Although reduction in hippocampal volume as assessed by MRI has been reported for the KA model (15, 28) and PILO (12, 29), comparative studies are still lacking. A longitudinal MRI evaluation of volumetric changes ensures that each animal can serve as its own control. However, to investigate possible correlations between MRI and histological data, a number of animals was perfused at each time point. As consequence only few animals were followed along the whole study. In this way, the longitudinal evaluation was prejudiced by the lower number of animals. On the other hand, it is important to recognize that 70% of PILO-treated animals were considered to have hippocampal atrophy at 3 months post SE and that this number increased to 88% 3 months later (6 months after SE). In contrast, 90% of KA-treated animals already displayed hippocampal atrophy at 3 months post SE. Given the evidence that these two models promote hippocampal cell damage at different times and intensities (6), we emphasize that all animals had at least 90 min of SE before we attenuated the seizures and that all animals had spontaneous seizures prior the first image acquisition. Thus, although the reduction in hippocampal volume did not progress with time, the number of animals that showed a reduction in hippocampal volume in the PILO model increased from 3 to 6 months post SE. Such a subtle difference could only be detected in a comparative study.

Previous studies have demonstrated that the number of damaged or dead neurons is low in the chronic PILO model (30, 31). This is in agreement with our current findings demonstrating a relative stability in terms of cell loss in the chronic phase of both models. Although, there was a greater reduction in the numbers of CA1 and CA3 pyramidal cells in the KA group, these animals displayed fewer SRS than those in the PILO group, indicating that in addition to cell loss, other forms of functional plasticity among surviving neurons play important roles in the disease progression. Although the present study was restricted to the hippocampus and thus could not be extrapolated to other brain areas, this plasticity may be triggered by alterations in transcriptional, translational, and post-translational regulation as well as in the function of transcription factors, ion channels (32), neurotransmitter receptors (33–35), transporters, and kinases (35–37) that may still occur in hippocampal neurons months after SE.

Similar to volumetric changes, T_2_ relaxation times showed subtle variations between the models studied that could only be detected because the experiments were performed in the animals under similar experimental conditions. The current results demonstrating hippocampal tissue reduction and increased T_2_HP are consistent with findings from human studies showing that SRS, MFS, hippocampal volume, and T_2_HP are not strongly correlated (38–47). This may suggest that similar alterations in other brain areas may be more relevant to epileptic outcome than those in the hippocampus; the piriform, and entorhinal cortices (13) are currently under investigation.

Increased T_2_ relaxation time may reflect a regional reduction in a cell population, which may lead to a decrease in water compartmentalization and, consequently, an increase in the amount of free water. Accordingly, a similar SE duration has been shown to cause more extensive hippocampal cell damage in the PILO than in the KA model (6), which may lead to an increase in the T_2_ relaxation time in the PILO group 3 months after SE. Although this explanation is not conclusive, as no correlation has been established between T_2_HP changes and cell loss, the expression of aquaporin 4, and T_2_ relaxation time are increased in the hippocampal region after pilocarpine-induced status (17) and in sclerotic hippocampus in human TLE (48). Other factors, as astrogliosis may contribute to T_2_HP changes and should be addressed in future studies, as recently reported in patients with mesial temporal epilepsy (49).

Longitudinal MRI is one strategy used to evaluate chronic epileptic patients. One study (50) has examined volume and T_2_HP changes in patients with TLE at two time points with an intervening interval of three and a half years. The initial comparison between patients with epilepsy and age-matched controls demonstrating hippocampal atrophy in the individuals with epilepsy was followed in the second evaluation by an increased T_2_HP signal and a further reduction in hippocampal volume. It is interesting to note that patients with or without significant volume reduction were comparable in terms of seizure frequency, antiepileptic drug (AED) use, and epilepsy duration, with no identifiable risk factors for the development of atrophy. Similar to our present results, none of the 14 patients with epilepsy and hippocampal atrophy in that study showed a further reduction in volume or T_2_HP over time. These results indicate that once detected, hippocampal atrophy remains stable, despite seizure frequency.

One limitation of the current study is that spontaneous seizures were not continuously monitored. One could suggest that the absence of correlation between the number of seizures and volumetric changes is related to the current used method to sample seizure frequency. In this study, we have video monitored all animals 12 h a day, 5 days a week, along the 9 months after status. These hours were preferentially at light time (7 am–7 pm) based on that no differences were reported in terms of clustered seizures or circadian rhythm for both KA and PILO models (51, 52). However, despite that, it is still possible that some spontaneous seizures were missed along the study, so future studies with more extensive monitoring could produce different results.

## Conclusion

In conclusion, our results showed that MRI may be used to follow specific model-associated changes, such as hippocampal atrophy. The absence of a correlation of the MRI volumetry and relaxometry data with epileptic outcome is in agreement with clinical-based data in which individual neuropathological changes are not necessarily related to the frequency of spontaneous seizures.

## Conflict of Interest Statement

None of the authors has any conflict of interest to disclose. We affirm that we have read the Journal’s position on issues involved in ethical publication and affirm that this report is consistent with those guidelines. Clement Hamani is a consultant for St Jude medical.

## Abbreviations

CA1, Ammon’s horn, field 1; CA3, Ammon’s horn, field 3; CH, caudal hippocampus; CTL, control group; EEG, electroencephalographical recordings; DG, dentate gyrus; KA, kainic acid; MRI, magnetic resonance imaging; MSME, multi-slice multi-echo sequence; PILO, pilocarpine hydrochloride; RARE, rapid acquisition with refocused echoes sequence; RH, rostral hippocampus; ROI, region of interest; SE, status epilepticus; SRS, spontaneous recurrent seizures; TLE, temporal lobe epilepsy; T_2_HP, hippocampal relaxation time.
